# Hepatocyte expressed chemerin-156 does not protect from experimental non-alcoholic steatohepatitis

**DOI:** 10.1007/s11010-022-04430-3

**Published:** 2022-04-21

**Authors:** Rebekka Pohl, Laura Eichelberger, Susanne Feder, Elisabeth M. Haberl, Lisa Rein-Fischboeck, Nichole McMullen, Christopher J. Sinal, Astrid Bruckmann, Thomas S. Weiss, Michael Beck, Marcus Höring, Sabrina Krautbauer, Gerhard Liebisch, Reiner Wiest, Josef Wanninger, Christa Buechler

**Affiliations:** 1 Department of Internal Medicine I, Regensburg University Hospital, 93053 Regensburg, Germany; 2 Department of Pharmacology, Dalhousie University, Halifax, NS B3H 4R2 Canada; 3 Biochemistry Center Regensburg (BZR), Laboratory for RNA Biology, University of Regensburg, Regensburg, Germany; 4 Children’s University Hospital (KUNO), Regensburg University Hospital, 93053 Regensburg, Germany; 5 Institute of Clinical Chemistry and Laboratory Medicine, Regensburg University Hospital, 93053 Regensburg, Germany; 6 Department of Visceral Surgery and Medicine, University Inselspital, 3010 Bern, Switzerland

**Keywords:** STAT3, p38, Triglycerides, Hepatic stellate cells, Gene expression

## Abstract

Non-alcoholic steatohepatitis (NASH) is a rapidly growing liver disease. The chemoattractant chemerin is abundant in hepatocytes, and hepatocyte expressed prochemerin protected from NASH. Prochemerin is inactive and different active isoforms have been described. Here, the effect of hepatocyte expressed muChem-156, a highly active murine chemerin isoform, was studied in the methionine–choline deficient dietary model of NASH. Mice overexpressing muChem-156 had higher hepatic chemerin protein. Serum chemerin levels and the capability of serum to activate the chemerin receptors was unchanged showing that the liver did not release active chemerin. Notably, activation of the chemerin receptors by hepatic vein blood did not increase in parallel to total chemerin protein in patients with liver cirrhosis. In experimental NASH, muChem-156 had no effect on liver lipids. Accordingly, overexpression of active chemerin in hepatocytes or treatment of hepatocytes with recombinant chemerin did not affect cellular triglyceride and cholesterol levels. Importantly, overexpression of muChem-156 in the murine liver did not change the hepatic expression of inflammatory and profibrotic genes. The downstream targets of chemerin such as p38 kinase were neither activated in the liver of muChem-156 producing mice nor in HepG2, Huh7 and Hepa1-6 cells overexpressing this isoform. Recombinant chemerin had no effect on global gene expression of primary human hepatocytes and hepatic stellate cells within 24 h of incubation. Phosphorylation of p38 kinase was, however, increased upon short-time incubation of HepG2 cells with chemerin. These findings show that muChem-156 overexpression in hepatocytes does not protect from liver steatosis and inflammation.

**Supplementary Information:**

The online version contains supplementary material available at 10.1007/s11010-022-04430-3.

## Introduction

Non-alcoholic steatohepatitis (NASH) is the progressive form of non-alcoholic fatty liver disease (NAFLD) and is a health challenge throughout the world [1–3]. The global prevalence of NAFLD is about 30% in the Middle East and South America, 27% in Asia, 24% in North America and Europe, and 14% in Africa [4]. Liver biopsy is an important diagnostic tool for NASH diagnosis and about 53% of NAFLD patients had NASH from biopsy data [5, 6]. Experts proposed to rename NAFLD to metabolic-dysfunction associated liver disease (MAFLD) and exclusion of other liver diseases such as viral infection or alcohol abuse is no longer required [7]. The prevalence of NAFLD and MAFLD in a representative study group of the US population was comparable as was the risk of advanced liver fibrosis [8].

Overweight is a risk factor for NASH and prevalence of NAFLD in Europe was 25% in normal-weight, 67% in overweight and 91% in the obese [4]. Adipose tissues secrete numerous proteins and metabolites which have an eminent role in metabolic liver diseases [1, 2].

Chemerin is an adipokine with multiple functions, and serum levels are increased in obesity [9, 10]. Chemerin can improve and worsen insulin resistance. This adipokine further functions as a pro- or anti-inflammatory molecule. Moreover, tumor-promoting and tumor-suppressive activities of chemerin were reported [9–12].

So far, two functional chemerin receptors have been identified, chemokine like receptor 1 (CMKLR1) and G protein-coupled receptor 1 (GPR1). C–C chemokine receptor-like 2 (CCRL2) is a non-signaling chemerin receptor, and increases local chemerin concentrations [10, 13, 14]. Activation of extracellular regulated kinase (ERK), Akt, p38 kinase and AMP-activated protein kinase is induced by chemerin binding to its receptors [11, 12, 15]. An early event in CMKLR1 and GPR1 signaling is the recruitment of beta-arrestin 2, which can be quantified by Tango assays [14, 16]. Phosphorylation of ERK by chemerin requires beta-arrestin 2 and G-proteins [14]. RhoA and rho-associated protein kinase are signaling molecules downstream of CMKLR1 and GPR1 [17].

Low concentrations of recombinant chemerin transiently increased the phosphorylation (p) of ERK in mature adipocytes. Higher levels of recombinant chemerin had less of an effect [18]. Activation of Akt by chemerin in hepatocytes was observed upon short incubation times. Exposure to recombinant chemerin for about two hours suppressed pAkt, and levels normalized about two hours later [15]. These observations suggest that cells become resistant to chemerin upon chronic exposure to high protein levels [15]. Thus, local chemerin concentrations, abundance of chemerin receptors and exposure time affect the biological functions of chemerin [10, 14, 19].

Cells secrete the inactive prochemerin, which has to be processed to function as a ligand for the chemerin receptors. The most active murine isoform is muChem-156 corresponding to human huChem-157. Shorter chemerin variants have a lower activity or are not active at all and may even inhibit active chemerin isoforms [10]. Accordingly, most of the data published so far describe the function of muChem-156/huChem-157 [10, 19].

Although chemerin is highly expressed in the liver, the physiological function of chemerin in liver cells has not yet been finally resolved [20]. Overexpression of huChem-157 in hepatocyte cell lines had no effect on cell proliferation and viability. ERK and p38 kinase were not activated [21]. This isoform increased migration of murine and human cell lines when overexpressed [15, 21].

Hepatic stellate cells have a central role in fibrogenesis [22, 23]. The human hepatic stellate cells LX-2 overexpressing huChem-157 did not differ from the control transfected cells regarding cell viability, proliferation, and expression of inflammatory and fibrogenic molecules [24].

Although current in-vitro data argue against a role of the most active chemerin variant in NASH, a previous study described a protective effect of huChem-157 in a murine model. Here, intraperitoneal injection of recombinant huChem-157 for 2 weeks in mice fed a high fat diet for 16 weeks improved hepatic steatosis, inflammation and liver injury [25]. These beneficial effects were prevented by inhibition of CMKLR1. Moreover, injection of active chemerin enhanced janus kinase 2—signal transducer and activator of transcription 3 (JAK2-STAT3) signaling, and blockage of this pathway impeded the effect of chemerin. In this model, recombinant chemerin reduced hepatic lipid levels. Serum triglyceride levels as well as very low density lipoproteins were increased and it was assumed that chemerin injection stimulated hepatic triglyceride release. Notably, chemerin injection lowered body weight in this model, and reduced levels of circulating leptin and IL-6 levels suggest a decrease of fat mass. Obesity is a main driver of liver steatosis [1, 2], and reduced adiposity rather than direct hepatic effects of huChem-157 may at least in part explain less hepatic steatosis and inflammation of these mice [25].

On the other hand, it was also shown that overexpression of huChem-157 promoted lipid accumulation in HepG2 cells. Expression of enzymes for fatty acid synthesis and oxidation were reduced in the HepG2 cells producing huChem-157. Interestingly, overexpression of prochemerin increased cellular triglyceride levels as efficient as huChem-157 [26].

Feeding rodents a methionine-choline deficient (MCD) is a model of NASH that induces hepatic steatosis and inflammation [27]. Overexpression of prochemerin in the murine liver reduced oxidative stress and hepatic macrophage load of MCD diet fed mice. There was, however, no effect of prochemerin on body weight, fat mass, serum glucose, insulin levels, hepatic triglyceride and cholesterol concentrations [28]. Notably, when human peripheral blood mononuclear cells (PBMCs) were cultivated in media of Huh7 cells overexpressing prochemerin, CC-chemokine ligand 2 (CCL2), interleukin-6 (IL-6), and osteopontin levels declined [28]. The conditioned medium of Huh7 cells overexpressing huChem-157 was ineffective in this regard [21].

So far there is no clear evidence for a direct hepatic effect of muChem-156/huChem-157 in NAFLD. The in-vitro data argue against a prominent role of this chemerin isoform in the liver [21, 24]. Moreover, in-vitro studies mostly used hepatocyte cell lines rather than primary cells [21, 24, 26] and strikingly differences between primary cells and tumor cells exist [29].

Aim of this study was to test the hypothesis that mice with hepatic overexpression of muChem-156 are not protected from NASH. Mice fed the MCD diet develop liver steatosis and inflammation [27, 30]. In MCD diet fed mice, an upregulation of hepatic chemerin protein was noticed. These animals lose body fat and this minimizes the impact of adipose tissue derived chemerin [31]. Hepatic overexpression of muChem-156 was achieved by infecting mice with recombinant adeno-associated virus 8 (AAV8) particles utilizing the mouse alpha-fetoprotein enhancer and the mouse minimal albumin promoter to overexpress chemerin in hepatocytes [32].

## Materials and methods

### Quantification of lipids

Triglycerides (Triglyceride Quantification Kit; BioVision, Milpitas, CA, USA, order number: K622-100-BV) and cholesterol (Diaglobal, Berlin, Germany, order number: CHO 013) of the murine liver and of hepatocytes incubated with recombinant chemerin were measured by commercial assays. Triglyceride and cholesterol levels of hepatocyte cell lines overexpressing chemerin were determined by mass spectrometry analysis [33, 34].

### Lactate dehydrogenase measurement and ELISAs

Lactate dehydrogenase (LDH) in the supernatants was analyzed by the Cytotoxicity Detection Kit from Roche (Mannheim, Germany, order number: 11644793001). Adiponectin and chemerin ELISAs were from R&D Systems [Wiesbaden, Nordenstadt, Germany, order numbers: DY1119, DY2325 (mouse) and DY2324 (human)].

### Animal studies

Male C57BL/6J mice from Charles River (Sulzfeld, Germany) were housed in a 21 ± 1 °C controlled room under a 12 h light–dark cycle. Animals had free access to food and water and 4 to 7 mice were housed per cage. Adeno-associated virus 8 (AAV8) (Sirion Biotech; Planegg-Martinsried, Germany) to overexpress muChem-156 (n = 8) or empty vector (n = 8) were applied to 9 week old male mice by intraperitoneal injection (10^11^ particles per animal). After injection of AAV8, the mice were fed a MCD diet (E15653-94) or the respective control diet (E15654-04, Ssniff, Soest, Germany) for 2 weeks.

This dietary model was used in our group before [35, 36]. Hepatic expression of F4/80, CD68, TNF, and TGFβ mRNA were induced and triglycerides accumulated in the liver. These mice do not develop liver fibrosis, and collagen1A1 (Col1a1) as well as alpha-smooth muscle actin (SMA) mRNA were not increased [35, 36]. Mice were killed by CO_2_ asphyxiation and subsequent cervical dislocation.

### Immunoblotting and histology

Immunoblotting protocol and method for Hematoxylin–Eosin and Sirius Red staining were already published [37, 38]. Antibodies used in the current experiments are listed in Supporting Table S1.

### Mass spectrometry of chemerin protein

Mass spectrometry method used to identify chemerin isoforms in the liver was recently described [32].

### Monitoring of gene expression by real-time RT-PCR

Real-time RT-PCR methodology was done as described [37] and primers used in the assays are listed in Supporting Table S2.

### Primary human cells and cell lines

Human liver tissue was from liver resections of patients undergoing partial hepatectomy for metastatic liver tumors of colorectal cancer. Hepatocytes were isolated by a modified two-step EGTA/collagenase perfusion procedure [39, 40] and hepatic stellate cells were purified from the remaining supernatants [40]. Primary human cells were treated with huChem-157 (R&D Systems, order number: 2324-CM-025) for 24 h in serum-free medium. The hepatocyte cell lines were cultivated as described [40].

### Portal and hepatic vein blood

Portal and hepatic vein blood of 8 patients (3 females and 5 males, age was 49 (40–76) years) with liver cirrhosis was analyzed. Model for end-stage liver disease score was 6 (6–13) and C- reactive protein was 4 (2–53) mg/l.

### GeneChip analysis

Total RNA was isolated from primary human hepatic stellate cells and primary human hepatocytes of three different donors. Cells were cultivated in medium supplemented with 0, 360, 480 and 600 ng/ml huChem-157 for 24 h. Sample processing and Affymetrix microarray hybridization (Human Gene 2.1 ST Array) were carried out at a genomics core facility: Center of Excellence for Fluorescent Bioanalytics (KFB, University of Regensburg, Germany). Transcriptome Analysis Console (Thermo Fisher Scientific, Schwerte, Germany) was used for Principal Component Analysis. GEO accession number is GSE156698.

### Tango assay

Ex-vivo activation of CMKLR1 and GPR1 by serum chemerin was analyzed by the Tango Assay [41].

### Recombinant expression of chemerin isoforms in hepatocytes

Recombinant expression of huChem-157 in HepG2 and Huh7 cells and of muChem-156 in Hepa1-6 cells was already reported [21].

### Statistical analysis

Data are given as boxplots. Statistical differences were analyzed by two-tailed Mann–Whitney U Test or paired t-test (SPSS Statistics 25.0 program). A value of p < 0.05 was regarded as significant.

## Results

### MuChem-156 expression in the liver increases hepatic but not systemic chemerin levels and activity

**Fig. 1:**
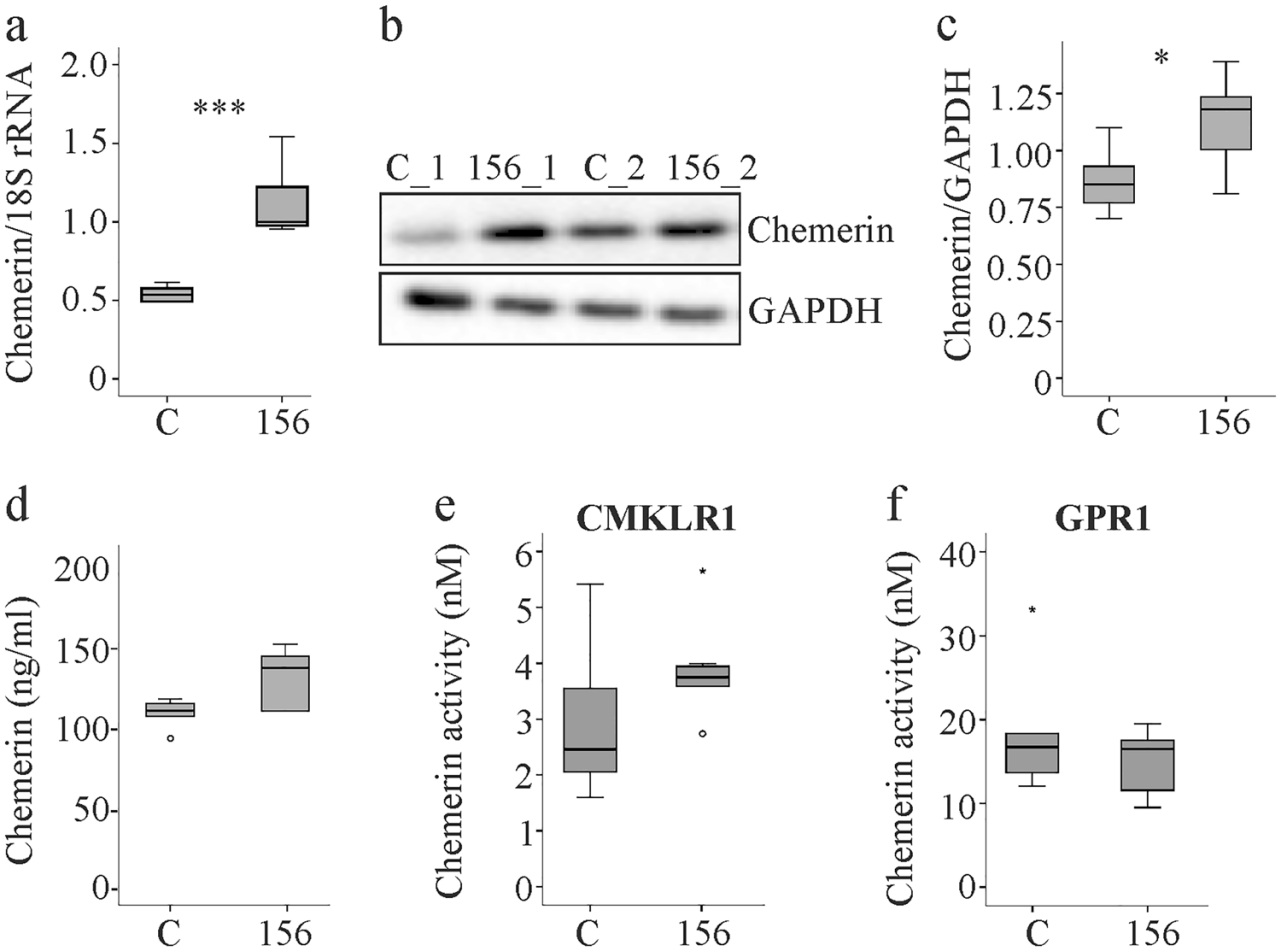
Overexpression of muChem-156 in the liver had no effect on serum chemerin and activation of chemerin receptors. **a** Chemerin mRNA in the liver of mice infected with control AAV8 (C) and mice infected with muChem-156 producing AAV8 (156). **b** Immunoblot of hepatic chemerin protein of two mice each. **c** Quantification of hepatic chemerin protein. **d** Serum chemerin. **e** Serum activation of CMKLR1. **f** Serum activation of GPR1. *p < 0.05, ***p < 0.001

MuChem-156 is a highly active murine chemerin isoform [10] that was overexpressed in the liver by infecting mice with AAV8 particles. Control animals were injected with empty virus that lacked a cloned cDNA. Immediately after virus injection, chow was switched to a methionine-choline deficient (MCD) diet for 2 weeks prior to sacrifice. Hepatic chemerin mRNA increased about 2-fold and protein about 1.5-fold in AAV8-muChem-156 infected mice (Fig. 1a–c). Serum chemerin, and accordingly, ex-vivo measured recruitment of beta-arrestin 2 to CMKLR1 and GPR1 by serum chemerin, did not change with hepatic muChem-156 overexpression (Fig. 1d–f). Liver produced muChem-156 thus did not add to systemic chemerin levels and activity with regard to the beta-arrestin 2 pathways. Moreover, mass spectrometry could not detect muChem-156 in the murine liver suggesting rapid degradation of this isoform (Supporting Fig. 1).

### The human liver releases inactive chemerin

**Fig. 2:**
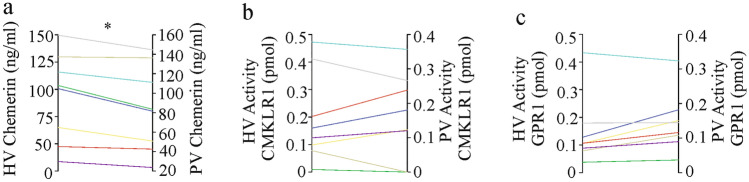
Chemerin protein levels, CMKLR1 and GPR1 activation in the hepatic vein (HV) and portal vein (PV). **a** Chemerin was measured by ELISA in PV and HV of eight patients with liver cirrhosis. **b** CMKLR1 activation of HV and PV blood. **c** GPR1 activation of HV and PV blood. *p < 0.05

Chemerin concentrations were higher in the hepatic vein than the portal vein of patients with liver cirrhosis [42]. Thus, the human liver releases chemerin into the circulation. To evaluate whether chemerin secreted by the human liver is active, blood was collected from the portal vein and the hepatic vein. For ethical issues, these samples were obtained from patients with liver cirrhosis during the insertion of a transjugular intrahepatic portosystemic shunt*.* Chemerin protein was higher in the hepatic than the portal vein blood (Fig. 2a). Activation of CMKLR1 or GPR1 by hepatic vein blood did not increase in parallel (Fig. 2b, c). This suggests that chemerin released by the human liver is inactive at least with respect to the beta-arrestin 2 pathway.

### MuChem-156 overexpression in the liver does not change body composition and hepatic steatosis

**Fig. 3:**
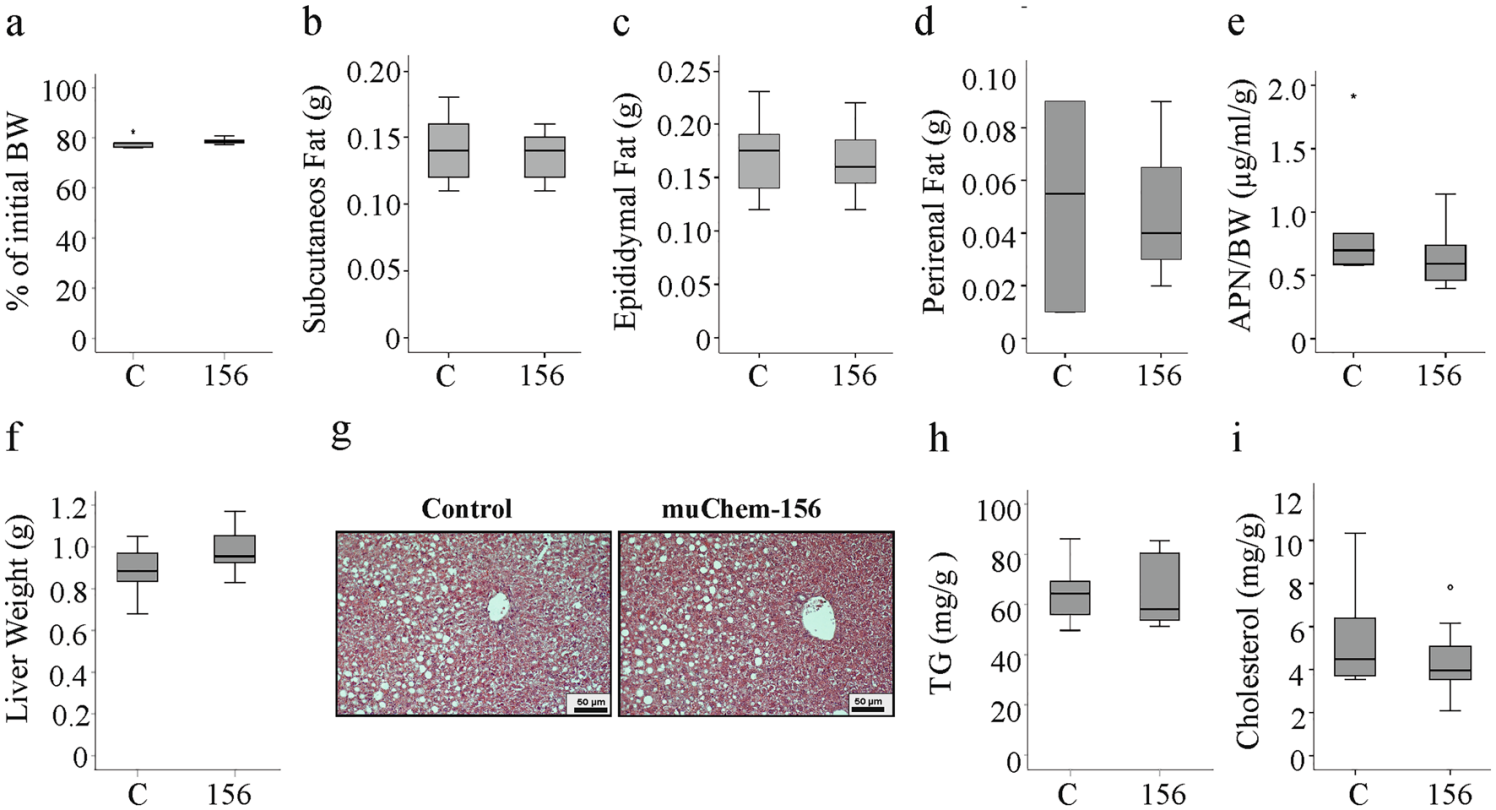
MuChem-156 overexpression had no effect on fat mass, circulating adiponectin and liver lipids. **a** Body weight (BW) at the end of the study relative to the initial weight in %. **B** Subcutaneous fat pad weight. **c** Epididymal fat pad weight. **d** Perirenal fat pad weight; **e** Serum adiponectin (APN) normalized to BW. **f** Liver weight; **g** Hematoxylin and Eosin stained liver. **h** Hepatic triglyceride (TG) levels. **i** Hepatic cholesterol levels

Mice overexpressing muChem-156 lost body weight as the control-treated animals (Fig. 3a). Accordingly, subcutaneous, epididymal and perirenal fat pad weights, and serum adiponectin levels were similar between the two groups (Fig. 3b–e). Liver weight, liver histology and levels of hepatic triglycerides and cholesterol were unaffected by muChem-156 (Fig. 3f–i).

### MuChem-156 expression in the liver does not activate already described chemerin-responsive signaling pathways

**Fig. 4:**
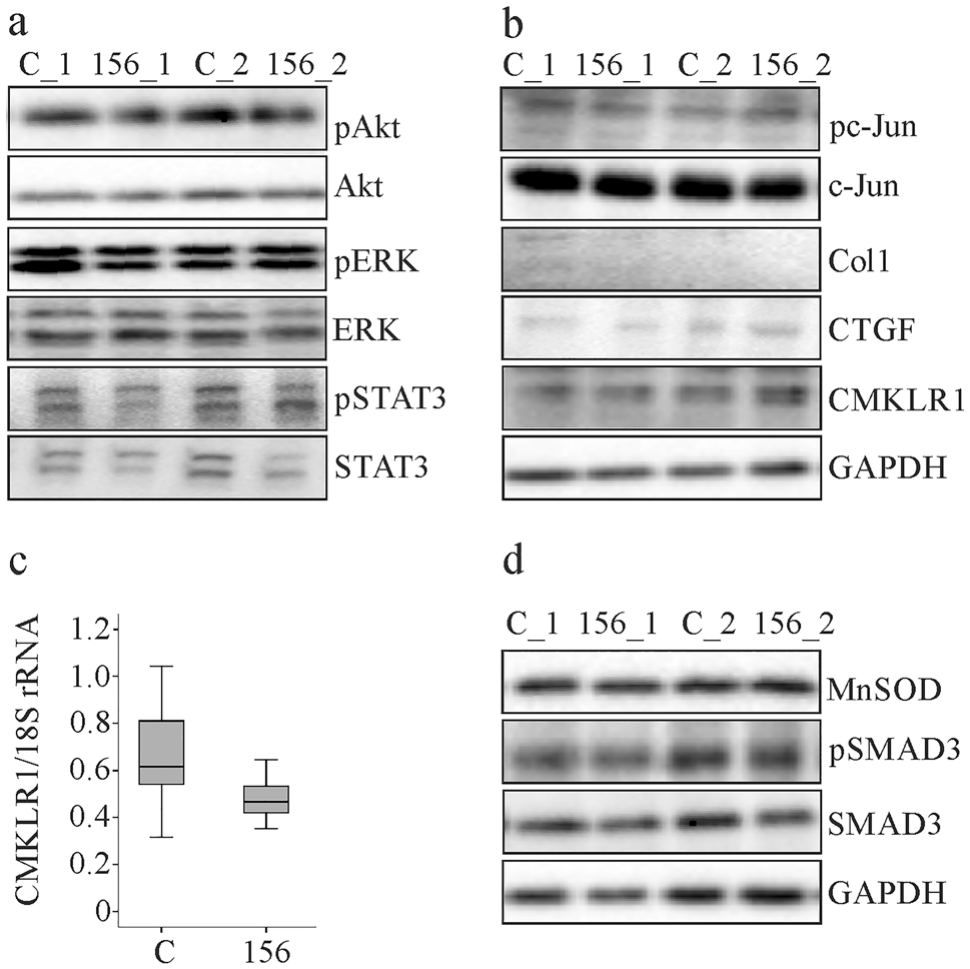
Expression of central signaling molecules, and makers of liver fibrosis in mice with muChem-156 overexpression. **a** Immunoblot analysis of AKT, ERK, STAT3 and their phosphorylated (p) forms. **b** Immunoblot analysis of c-Jun, pc-Jun, Col1, CTGF and CMKLR1. **c** CMKLR1 mRNA in the liver of controls and muChem-156 overexpressing mice. **d** Immunoblot analysis of MnSOD, pSMAD3 and SMAD3

Signaling molecules recently described to be activated by muChem-156 (Akt, ERK and STAT3 [12, 25]) were comparable between the groups (Fig. 4a). NASH is associated with higher expression of c-Jun and pc-Jun [43]. Both of these molecules were not affected by muChem-156 overexpression further excluding an effect of muChem-156 on liver disease severity (Fig. 4b). Sirius Red staining revealed that 2 weeks feeding of a MCD diet does not induce liver fibrosis [36], and accordingly, collagen and connective tissue growth factor (CTGF) were hardly detectable in the liver (Fig. 4b).

CMKLR1 is involved in the upregulation of ERK by recombinant chemerin [14] and, therefore, it was investigated whether this receptor is still expressed in the NASH liver of the MCD diet fed mice. CMKLR1 mRNA and protein were expressed in the liver and were not modified by muChem-156 overexpression (Fig. 4b, c).

The mitochondrial enzyme manganese superoxide dismutase (MnSOD) is reduced in NASH liver [44] and was similarly expressed in both groups. Phosphorylated mothers against decapentaplegic homolog 3 (SMAD3), a key mediator of fibrosis [45], was not changed upon muChem-156 overexpression (Fig. 4d).

### MuChem-156 expression did not change mRNA expression of inflammatory and fibrotic genes in the liver

MuChem-156 expression did not protect the animals from NASH. Indeed, hepatic mRNA levels of the chemokines CCL2, 3, 5, and 7, of the macrophage expressed genes F4/80, cluster of differentiation (CD)163, CD38 and CD68, of the cytokines IL-6 and tumor necrosis factor (TNF), and of Ly49C and natural cytotoxicity triggering receptor 1 (Ncr1), with the latter two being expressed by natural killer cells, were not changed with muChem-156 overexpression (Table 1). Transforming growth factor (TGF)-beta, alpha-smooth muscle actin (SMA) and collagen (Col)1a1 mRNA levels were also comparable across both groups (Table 1).

**Table 1:** Median values and range of genes expressed in the liver of control and muChem-156 overexpressing mice after feeding a MCD diet for 2 weeks

	Control	muChem-156
Alpha-SMA	0.84 (0.45–2.37)	0.68 (0.59–0.94)
CCL2	0.39 (0.28–1.28)	0.51 0.34–1.03)
CCL3	0.28 (0.17–0.31)	0.27 (0.17–0.63)
CCL5	0.50 (0.43–0.65)	0.53 (0.32–0.85)
CCL7	0.13 (0.09–0.48)	0.18 (0.10–0.32)
CD38	0.54 (0.35–0.72)	0.44 (0.32–0.86)
CD68	0.49 (0.33–1.16)	0.66 (0.41–1.08)
CD163	0.54 (0.26–1.06)	0.47 (0.30–0.72)
Col1a1	0.69 (0.34–1.17)	0.65 (0.38–1.32)
F4/80	0.90 (0.76–1.36)	0.89 (0.37–2.25)
IL-6	0.56 (0.44–0.67)	0.43 (0.22–0.71)
Ly49C	0.51 (0.39–0.80)	0.41 (0.22–0.68)
Ncr1	0.63 (0.47–0.72)	0.43 (0.29–1.07)
TGF-beta	0.85 (0.53–0.98)	0.61 (0.39–0.90)
TNF	0.76 (0.30–1.32)	0.87 (0.18–1.05)

### Recombinant huChem-157 had no effect on cell viability and gene expression of primary human hepatocytes and hepatic stellate cells

**Fig. 5:**
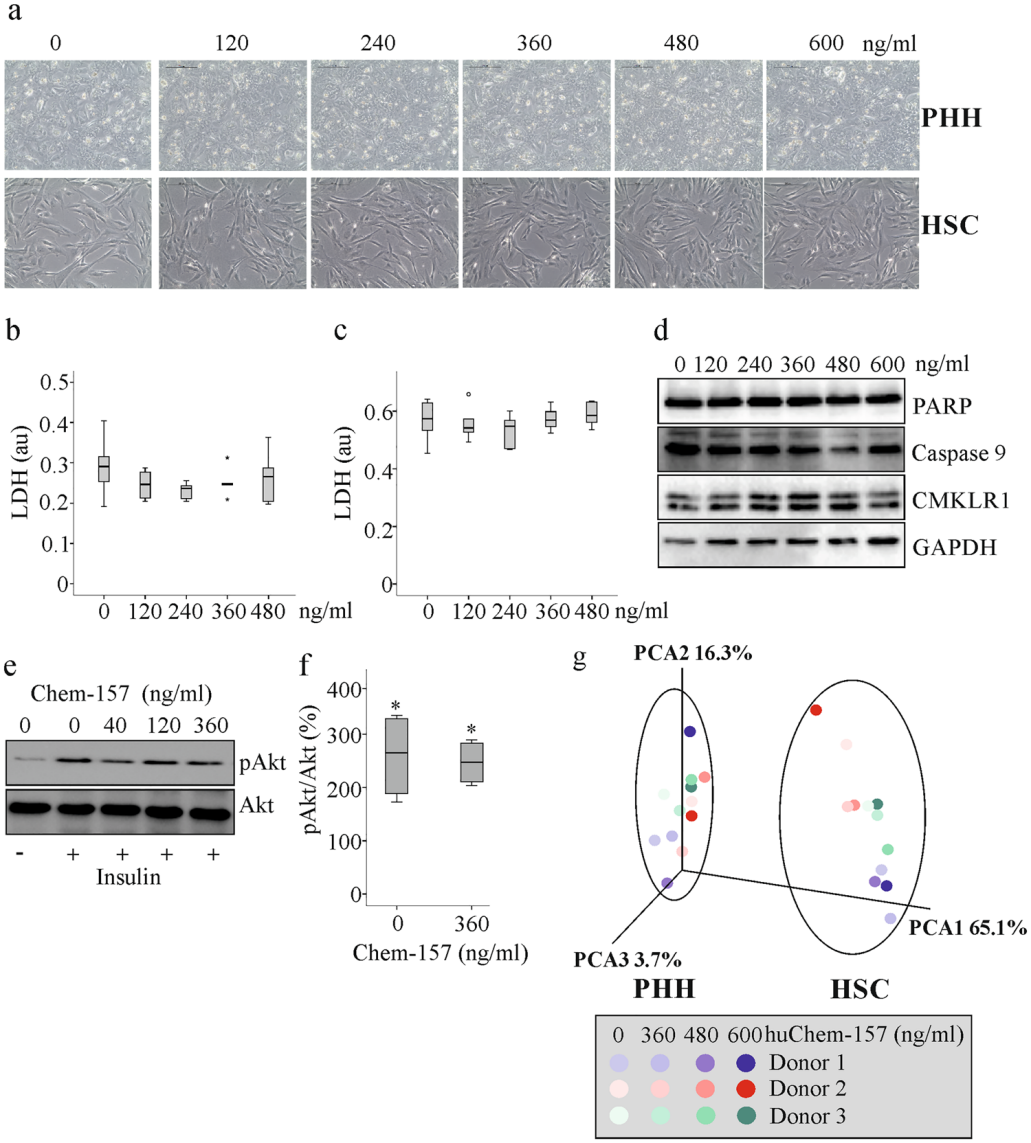
HuChem-157 had no effect on global gene expression and viability of primary human hepatic stellate cells (HSC) and primary human hepatocytes (PHH). Insulin signaling of PHH is not impaired by huChem-157. **a** Microscopy of PHH and HSC cultivated for 24 h in the presence of increasing concentrations of recombinant huChem-157. **b** LDH in the supernatant of PHH (n = 6). **c** LDH in the supernatant of HSC (n = 6). **d** Immunoblot of PARP, caspase 9 and CMKLR1 of HSC cultivated for 24 h in the presence of increasing concentrations of recombinant huChem-157. **e** PHH were pre-incubated with huChem-157 for 24 h and, 30 min after addition of insulin, pAkt was analyzed by immunblot. **f** pAkt/Akt ratio (% relative to non-insulin incubated cells) in PHH preincubated with 0 or 360 ng/ml huChem-157 for 24 h and stimulated with insulin. **g** Principle component analysis (PCA) of PHHs and HSCs cultivated for 24 h in the presence of increasing concentrations of recombinant huChem-157

Current in-vivo data suggest a minor if any role of muChem-156 in the liver. To further strengthen this suggestion, the effect of huChem-157 was analyzed in human hepatocytes and hepatic stellate cells. HuChem-157 is the human homolog of muChem-156 and the best studied bioactive human chemerin isoform to date [10]. Increasing concentrations of recombinant huChem-157 (120, 240, 360 and 480 ng/ml) had no effect on cell morphology and cell viability (which was analyzed by measuring lactate dehydrogenase levels in cell media) of primary human hepatocytes and hepatic stellate cells (Fig. 5a–c). Similarly, huChem-157 did not induce apoptosis of primary human hepatic stellate cells as evidenced by an absence of poly-(ADP-ribose) polymerase and caspase 9 cleavage (Fig. 5d). CMKLR1 is expressed in hepatic stellate cells and this was shown before [31], and is not regulated by treatment with huChem-157 (Fig. 5d).

Chemerin regulates insulin response of adipocytes [10]. Preincubation of primary human hepatocytes with up to 360 ng/ml recombinant huChem-157 for 24 h did, however, not affect insulin-induced (200 nM insulin for 30 min) Akt phosphorylation (Fig. 5e, f).

Gene Chip analysis did not identify any differentially regulated gene in primary human hepatocytes and primary human hepatic stellate cells, which were cultivated in the presence of 360, 480 or 600 ng/ml huChem-157 for 24 h. This was illustrated by principal component analysis where hepatic stellate cells and primary human hepatocytes (3 different donors each) were well separated but huChem-157 did not cause a common shift in gene expression (Fig. 5g).

### Recombinant huChem-157, but not overexpression of active chemerin, activates ERK, p38 MAPK and STAT3

**Fig. 6:**
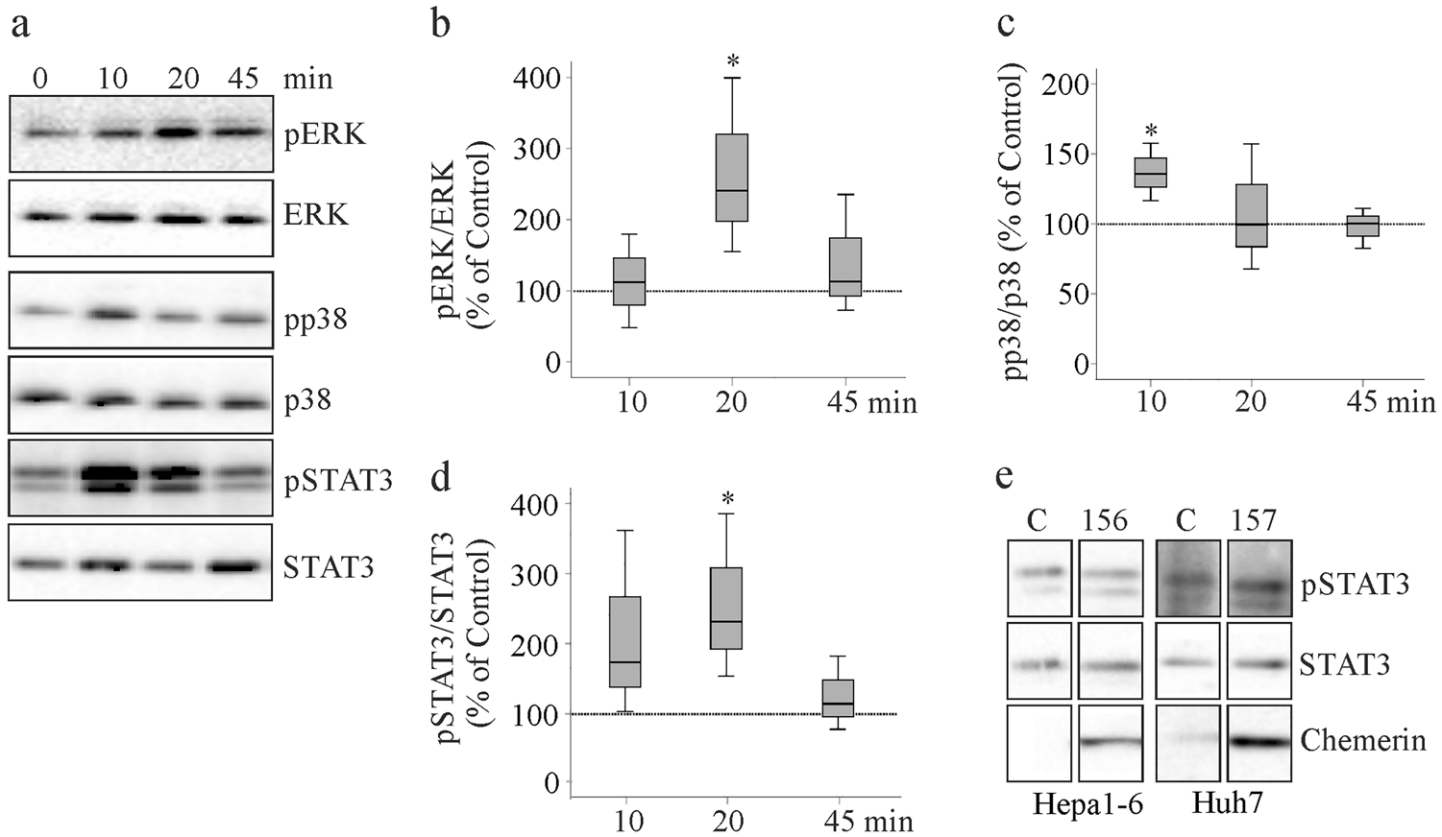
Activation of ERK, p38 kinase and STAT3 by recombinant chemerin. **a** Immunoblot analysis of ERK, p38 kinase, STAT3 and their phosphorylated (p) forms after incubation with 600 ng/ml recombinant huChem-157 for the indicated times. **b** Quantification of pERK/ERK. **c** Quantification of pp38/p38 kinase. **d** Quantification of pSTAT3/STAT3. **e** Immunoblot analysis of STAT3 and pSTAT3 in Hepa1-6 and Huh7 cells overexpressing active chemerin. *p < 0.05 (n = 3)

Previous studies described activation of ERK, p38 kinase and STAT3 by huChem-157/muChem-156 [12, 25]. Recombinant huChem-157 (600 ng/ml) activated ERK in HepG2 cells after 20 min incubation time and levels normalized at 45 min (Fig. 6a, b). Increased phosphorylation of p38 kinase was observed after 10 min treatment, and returned to the basal levels at 20 and 45 min (Fig. 6a, c). Phosphorylation of STAT3 at Y705 was increased by 10 and 20 min incubation with huChem-157, at 45 min this effect disappeared (Fig. 6a, d).

When huChem-157 was overexpressed in HepG2 cells or Huh7 cells, ERK and p38 kinase were not activated [21]. This also applied to Hepa1-6 cells overexpressing muChem-156 [21]. STAT3 phosphorylation was not induced in Hepa1-6 cells, Huh7 cells or HepG2 cells with overexpression of active chemerin (Fig. 6e and data not shown).

Chemerin was measured by ELISA and was 105 (71–190) ng/ml in HepG2 media and 54 (32–57) ng/ml in Huh7 cell media of huChem-157 overexpressing cells [21]. Chemerin in Hepa1-6 cell media was 201 (118–257) ng/ml [21]. To be able to compare chemerin concentrations in cell media with the amount of recombinant huChem-157, an ELISA was performed. This analysis showed that 100 ng/ml recombinant protein is equivalent to 25 ng/ml as determined by ELISA. The 600 ng/ml recombinant protein used for stimulation of HepG2 cells (Fig. 6a) is comparable to 150 ng/ml in cell media.

### Recombinant huChem-157 and overexpression of active chemerin had no effect on cellular lipids

Recent studies identified an effect of huChem-157 on hepatic lipid levels in mice and in HepG2 cells [25, 26]. Mass spectrometry was used to quantify cellular triglycerides, cholesteryl ester and free cholesterol in Hepa1-6 cells, HepG2 cells and Huh7 cells overexpressing active chemerin. This analysis showed that Hepa1-6 cells have a lot less triglycerides and cholesteryl esters per mg cellular protein than the human cell lines. There was no effect of chemerin overexpression on the levels of these lipids in any cell line (Table 2).

**Table 2:** Triglyceride, cholesteryl ester, free cholesterol and total cholesterol (which is the sum of cholesteryl ester and free cholesterol) in Hepa1-6 cells, Huh7 cell and HepG2 cells transfected with the corresponding control plasmid (control) or a plasmid to express muChem-156 or huChem-157

	Triglycerides (nmol/mg protein)	Cholesteryl ester (nmol/mg protein)	Free cholesterol (nmol/mg protein)	Total cholesterol (nmol/mg protein)
Hepa1-6				
Control	3.3 (2.0–6.5)	2.8 (1.8–4.8)	47.8 (28.4–66.5)	51.4 (30.2–69.9)
muChem-156	4.8 (4.5–5.2)	3.3 (2.8–4.4)	56.1 (51.7–70.5)	59.7 (55.0–70.5)
Huh7				
Control	91.9 (80.5–103.2)	27.5 (25.0–28.4)	73.6 (60.9–84.8)	101.1 (89.2–109.8)
huChem-157	86.6 (51.3–175.4)	25.5 (14.0–47.3)	69.4 (47.6–130.5)	94.9 (61.6–177.8)
HepG2				
Control	43.5 (39.3–73.7)	19.6 (9.7–31.2)	54.0 (22.0–85.3)	73.6 (31.7–116.5)
huChem-157	58.4 (31.1–135.2)	24.0 (10.8–30.6)	72.4 (35.7–86.0)	96.4 (46.6–116.6)

Median values, minimum and maximum are given (n = 4). There was no effect of chemerin overexpression on the levels of these lipids in any cell line

**Fig. 7:**
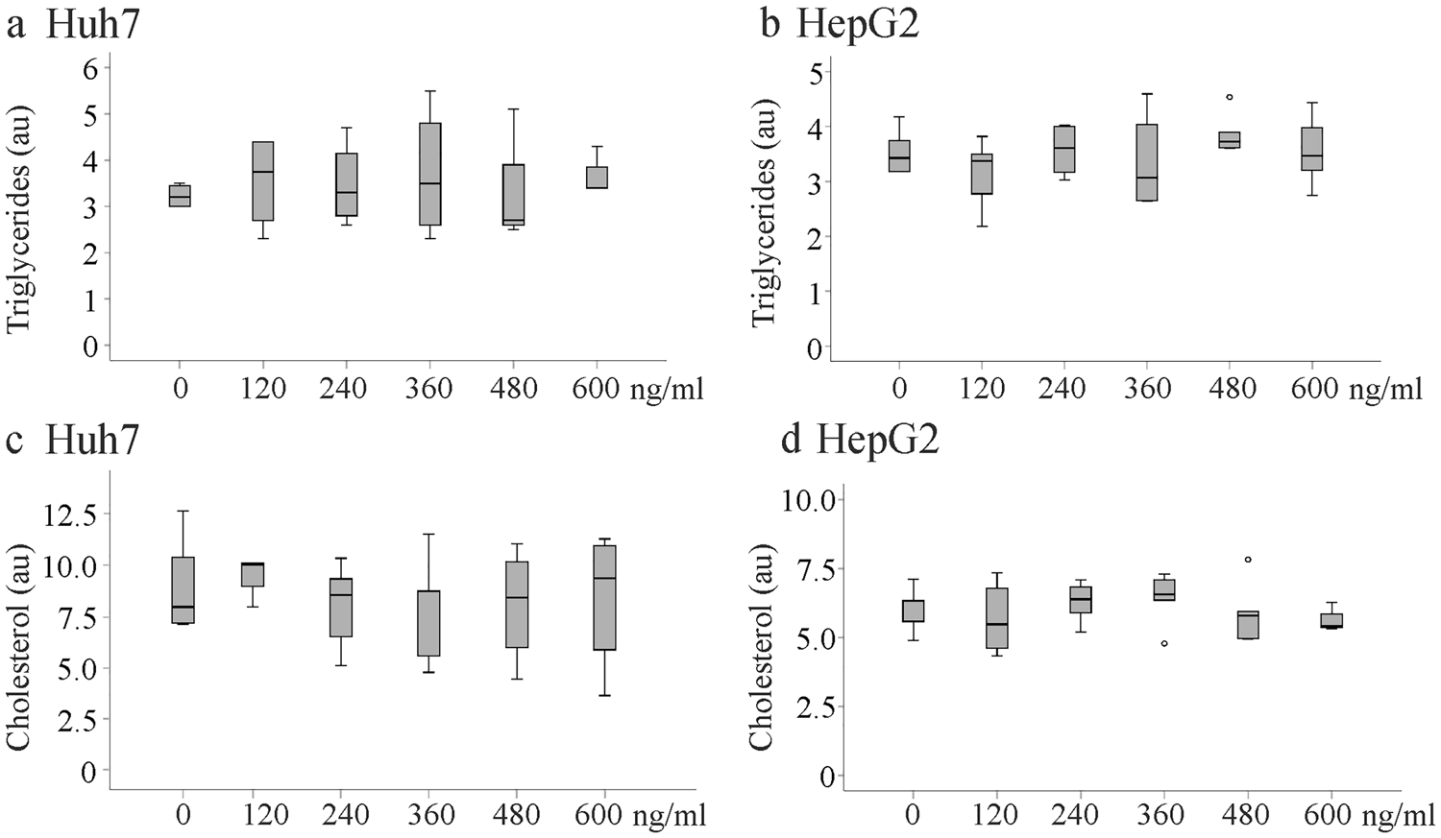
Cellular lipids of Huh7 and HepG2 cells cultivated for 24 h in the presence of increasing concentrations of recombinant huChem-157. **a** Triglyceride levels of Huh7 cells. **b** Triglyceride levels of HepG2 cells. **c** Cholesterol levels of Huh7 cells. **d** Cholesterol levels of HepG2 cells (arbitrary units, au)

When Huh7 cells and HepG2 cells were treated with increasing concentrations of recombinant huChem-157 (0, 120, 240, 360, 480, 600 ng/ml) for 24 h there was no change of cellular triglyceride and cholesterol levels (Fig. 7a–d).

## Discussion

Present data do not support a role for the most active chemerin isoform in NASH. Overexpression of muChem-156 in the liver did not affect hepatic triglyceride and cholesterol levels. A change in the expression of proinflammatory and profibrotic genes was not observed.

Accumulation of triglycerides and cholesterol is a characteristic of NAFLD, and also occurs in the liver of mice fed a MCD diet. In the present study it was shown that overexpression of muChem-156 did not change hepatic triglyceride and cholesterol levels. Accordingly, there was no effect of muChem-156/huChem-157 overexpression on cellular levels of triglycerides, cholesteryl ester and free cholesterol levels of three different hepatocyte cell lines. Treatment of HepG2 and Huh7 cells with recombinant huChem-157 did not change cellular triglyceride and cholesterol concentrations. A further study reported that injection of recombinant chemerin in apolipoprotein E deficient mice did not alter hepatic triglyceride and cholesterol levels [46]. Altogether, the present data do not provide evidence for a role of muChem-156/huChem-157 in hepatic lipid storage.

This is in contrast to a previous study showing that chemerin-156, -157, -158 and -163, when overexpressed in HepG2 cells, increased cellular triglyceride levels by 30 to 60%. Total chemerin protein in cell media of HepG2 cells overexpressing huChem-157 was about 2-fold higher in comparison to control transfected cells [26]. Notably, overexpression of CMKLR1 or GPR1 in the HepG2 cells led to an about 2.5-fold higher triglyceride content [26]. This suggests that chemerin produced by HepG2 cells increases cellular triglyceride levels and this is mediated by the chemerin receptors. The effect of the chemerin variants was exerted by GPR1 in this cell line. Indeed CMKLR1 mRNA could not be detected [26]. This is in contrast to other findings showing that CMKLR1 protein is expressed in HepG2 cells [15] whereas GPR1 mRNA could not be amplified [24].

CMKLR1 deficient mice are not protected from liver steatosis in experimental NASH [47]. It was also reported that CMKLR1 null mice had less liver steatosis but this was most likely related to the reduced adiposity of these animals [48]. Overexpression of prochemerin in the murine liver had no effect on hepatic triglyceride and cholesterol levels in MCD diet fed mice [28]. GPR1 loss did not change hepatic lipid accumulation of mice fed a normal chow or a high fat diet [49]. A separate study showed that intraperitoneal injection of human recombinant, active chemerin reduced the steatosis score of mice fed a high fat diet [25]. Here, hepatic chemerin protein of the chemerin treated mice was about 2-fold higher [25]. Notably, chemerin injection decreased food intake and body weight and this may secondarily lead to less hepatic steatosis.

Thus, currently there is no convincing evidence that chemerin or its receptors directly regulate hepatic lipid levels.

HepG2 cells are tumor cells, which have an altered lipid metabolism [50]. At the moment, there is no explanation why chemerin enhanced lipid deposition of these cells in one study [26] but had no effect in the current investigation. In the present study besides HepG2 cells, Huh7 and Hepa1-6 cells as well as primary human hepatocytes were employed. Recombinant chemerin did not alter cellular triglyceride and cholesterol levels of any cell type further excluding that chemerin has a role herein.

It has to be noted that cellular triglyceride and cholesterol levels of Hepa1-6 cells were rather low in comparison to HepG2 and Huh7 cells. Higher cellular triglyceride levels because of enhanced de novo lipogenesis were already reported for Huh7 cells in comparison to HepG2 cells [51] and this was confirmed in the current analysis. Cholesterol concentrations of the two human hepatocyte cell lines were rather similar. In the murine and human liver triglyceride levels are about 10 nmol/mg, cholesteryl ester about 1 nmol/ml and free cholesterol about 7 nmol/mg [35, 36, 52]. Thus, triglycerides of Hepa1-6 cells are about threefold lower, of Huh7 cells are about ninefold and of HepG2 cells about fourfold higher compared to the liver. Free cholesterol levels are about 5 to sevenfold higher in the cell lines, and cholesteryl ester levels are 3- to 27-fold higher. Thus, lipid composition of these three cell lines and the liver differs greatly. Fatty acids and/or cholesterol biosynthesis are increased in hepatocellular carcinomas [50] and, with the exception of low triglyceride levels in Hepa1-6 cells, these lipids accumulated in the hepatocyte cell lines and may be regarded as a characteristic of malignant cells.

Recent studies showed that recombinant muChem-156 had no effect on hepatocyte viability and proliferation [15, 21, 53, 54]. In agreement with these findings, recombinant huChem-157 did not exert cytotoxic effects in primary human hepatocytes. This also applied to human hepatic stellate cells. Indeed, global gene expression analysis of primary human hepatocytes and hepatic stellate cells did not identify any gene regulated in response to huChem-157 treatment. Furthermore, overexpression of muChem-156/huChem-157 in hepatocytes or muChem-156 in the liver had no effect on central signaling molecules [21]. The present study also showed that increasing doses of huChem-157 did not affect insulin-induced Akt phosphorylation in primary human hepatocytes. From these data we conclude that muChem-156/huChem-157 has no gross effects on hepatocytes and hepatic stellate cells. Accordingly, the severe liver damage in the diethylnitrosamine HCC model was unaltered by muChem-156 overexpression [32]. Of note, overexpression of muChem-156 could not improve hepatic inflammation in the murine NASH model studied herein.

One novel aspects of our study was that muChem-156 was overexpressed in the hepatocytes of mice. Moreover, a NASH model not accompanied by fat mass gain was used. It has to be noted that hepatocyte produced and adipocyte released chemerin seem to have different biological roles [55]. Whereas knock-down of hepatic chemerin had no effect on blood pressure, whole body knock-down of chemerin reduced blood pressure. Chemerin is most abundant in adipocytes and hepatocytes, making it likely that blood pressure is affected by adipocyte produced chemerin in the whole body knock-down model [55].

Present study further indicated that muChem-156 was rapidly processed in the NASH liver. This idea is supported by the observation that hepatic overexpression of muChem-156 neither changed serum chemerin protein levels nor serum activation of CMKLR1/GPR1. Preliminary analysis could not detect muChem-156 in the liver even in mice with muChem-156 overexpression.

Chemerin protein is abundant in the liver, and accordingly, chemerin protein was higher in the hepatic vein than the portal vein of patients with liver cirrhosis [42]. The capacity to activate CMKLR1 or GPR1 did not increase in parallel to chemerin protein levels. Altogether, these observations suggest that, similar to mice, chemerin derived from the human liver is inactive at least with regard to the activation of the beta-arrestin 2 pathway. Limitation of this analysis was that only eight patients were included. Moreover, for ethical issues samples were obtained from patients with liver cirrhosis. Further studies are needed to finally verify whether liver released chemerin is biologically inactive.

The activation of ERK, p38 kinase and STAT3 by muChem-156/huChem-157 was already reported [12, 25]. Short-time incubation with huChem-157 activated all of these molecules in HepG2 cells. Such an effect was not observed when muChem-156/huChem-157 were overexpressed in hepatocytes.

The in-vitro studies described above used up to 600 ng/ml recombinant chemerin, which was added to culture media of HepG2 cells. This corresponds to 150 ng/ml chemerin when determined by an ELISA. In media of hepatocytes overexpressing active chemerin 54, 105, and 201 ng/ml chemerin were detected by ELISA [21]. Thus, at least in the Hepa1-6 model, there was sufficient chemerin protein to reproduce the effects of the recombinant protein. Constant exposure to recombinant protein could make the cells resistant to the effects of chemerin, and this assumption has been made earlier [15, 18].

To sum up, the current work showed that modest overexpression of muChem-156 in the liver did not protect from NASH. In line with these in-vivo findings, neither overexpression of muChem-156/huChem-157 nor supplementation of cell media with recombinant huChem-157 had an effect on hepatocyte lipid storage. HuChem-157 did not modulate viability of primary liver cells or hepatocyte insulin response and did not change global gene expression of these cells. Present results show that the most active chemerin isoform does not greatly affect hepatocyte function and, when overexpressed in the liver, does not protect from NAFLD.

C-terminal peptides derived from chemerin, in particular the chemerin C_15_ peptide, are potent anti-inflammatory molecules and suppress inflammation through CMKLR1 [56]. Injection of this peptide did, however, not improve NASH of mice fed an atherogenic diet [57]. Thus it is unlikely that biologically active chemerin variants are hepatoprotective agents. Notably, overexpression of prochemerin reduced hepatic inflammation in experimental NASH and future studies are needed to characterize the molecular pathways involved. Clinical studies evaluating an association of hepatic prochemerin expression and liver injury may clarify whether high expression of prochemerin protects from disease progression. No pharmacological therapies are until now approved for NAFLD and weight loss is the recommended therapy [58]. Identification of the pathways involved in the protective effects of hepatocyte produced prochemerin may identify new strategies to treat NAFLD. Though current findings exclude a role of hepatocyte produced muChem-156 herein, injection of active chemerin isoforms may nevertheless improve NAFLD pathology by reducing body weight [25].

## Supplementary Information

Below is the link to the electronic supplementary material.

Supplementary file1 (DOCX 34 KB)

## Data Availability

All data generated are included in this article. Original data are available from the corresponding author on request.
