# Supplementary material for: Hepatocyte expressed chemerin-156 does not protect from experimental non-alcoholic steatohepatitis
Source: Mol Cell Biochem. 2022 Apr 21;477(8):2059–71. doi: 10.1007/s11010-022-04430-3 (PMC9237010; doi:10.1007/s11010-022-04430-3)

Hepatocyte expressed chemerin-156 does not protect from experimental non-alcoholic steatohepatitis

Rebekka Pohl ^1^, Laura Eichelberger ^1^, Susanne Feder ^1^, Elisabeth M. Haberl ^1^, Lisa Rein-Fischboeck ^1^, Nichole McMullen ^2^, Christopher J Sinal ^2^, Astrid Bruckmann ^3^, Thomas S. Weiss ^4^_,_ Michael Beck ^1,^ Marcus Höring ^5^, Sabrina Krautbauer ^1,5^, Gerhard Liebisch ^5^, Reiner Wiest ^6^, Josef Wanninger ^1^, Christa Buechler ^1, *^

**Supporting Table S1** Antibodies used in the present study.

| **Antigen** | **Company** | **Host** | **Order Number** |
| --- | --- | --- | --- |
| Akt | Cell Signaling | rabbit | 4691 |
| Caspase 9 | Calbiochem | rabbit | 218794 |
| Chemerin (human/mouse) | R&D Systems | goat | AF2324/5 |
| C-Jun | Cell Signaling | rabbit | 9258 |
| CMKLR1 | Abcam | rabbit | ab64881 |
| Collagen | Rockland | rabbit | 600-401-103-0.1 |
| CTGF | ThermoFisher | rabbit | BS-0743R |
| ERK | Cell Signaling | mouse | 9107 |
| GAPDH | Cell Signaling | rabbit | 2118 |
| MnSOD | Lab Frontier | rabbit | LF-PA0021 |
| p38 kinase | Cell Signaling | rabbit | 9212 |
| PARP | Cell Signaling | rabbit | 9542 |
| phospho-Akt (Ser473) | Cell Signaling | rabbit | 4060 |
| phospho-ERK (Thr202/Tyr204) | Cell Signaling | rabbit | 4370 |
| phospho-c-Jun | Cell Signaling | rabbit | 4668 |
| phospho-p38 kinase (Thr180/Tyr182) | Cell Signaling | rabbit | 4511 |
| phospho-Smad3 (Ser423/425) | Cell Signaling | rabbit | 9520 |
| phospho-STAT3 (Tyr705) | Cell Signaling | rabbit | 9145 |
| SMAD3 | Abcam | rabbit | ab28379 |
| STAT3 | Cell Signaling | mouse | 9139 |

**Supporting Table S2** Primers used for real-time PCR.

| **Gene** | **Primer uni 5´→ 3´** | **Primer rev 5´→ 3´** |
| --- | --- | --- |
| 18S rRNA | GATTGATAGCTCTTTCTCGATTCC | CATCTAAGGGCATCACAGACC |
| alpha-SMA | CCAGCACCATGAAGATCAAG | CTTCGTCGTATTCCTGTTTGC |
| CCL2 | AACTCTCACTGAAGCCAGCTCT | CTCTTGAGCTTGGTGACAAAAA |
| CCL3 | TGCCCTTGCTGTTCTTCTCT | GTGGAATCTTCCGGCTGTAG |
| CCL5 | TGCAGAGGACTCTGAGACAGC | GAGTGGTGTCCGAGCCATA |
| CCL7 | TTCTGTGCCTGCTGCTCATA | TTGACATAGCAGCATGTGGAT |
| CD163 | GTTGTGACCGCCTGTATGAG | TTCCCAACTAGCTTTTCACCTC |
| CD38 | ACGCTGCCTCATCTACACTC | GGGGCGTAGTCTTCTCTTGT |
| CD68 | TGATCTTGCTAGGACCGCTTA | CCCCTTGGACCTTGGACTA |
| Chemerin | AAACACCCACCTGTGCAGT | TTTTACCCTTGGGGTCCATT |
| CMKLR1 | cttctcccctaatcccctca | ggggtgagtgagccattt t |
| Col1a1 | CAGGGTCCTCCTGGTTCTC | GACCGTGAGTCCGTCTTTG |
| F4/80 | TGCTCTTCCTGATGGTGAGA | CCCCGTCTCTGTATTCAACC |
| IL-6 | CTCTGGGAAATCGTGGAAAT | CCAGTTTGGTAGCATCCATC |
| Ly49C | CCCTATTCCAGGGAGCTGT | TCTGTTTACCAGGAAGGAAGATG |
| Ncr1 | TTGGCTCTTACAACGACTATGC | GTTGAAAGGTCAAACTCCCAAT |
| TGF-β | CTGGGCACCATCCATGAC | CAGTTCTTCTCTGTGGAGCTGA |
| TNF | CCGATGGGTTGTACCTTGTC | GGGCTGGGTAGAGAATGGAT |

**Supporting Fig. 1** MuChem-156 isoform was not detected in the murine NASH liver. Mass spectrometry analysis was performed to identify chemerin isoforms in the liver. Protein was digested with trypsin, which cleaved chemerin after amino acid 136, 157 and 160. This approach is appropriate to identify muChem-156 and smaller isoforms but not prochemerin. Noteworthy, muChem-156 was not detected in the liver of control infected mice (analysis of 4 mice). Unexpectedly, muChem-156 was not identified in the liver of 3 mice overexpressing muChem-156. Though further analysis of hepatic chemerin isoforms is needed, these preliminary results suggest that muChem-156 seems to be a rare hepatic variant.


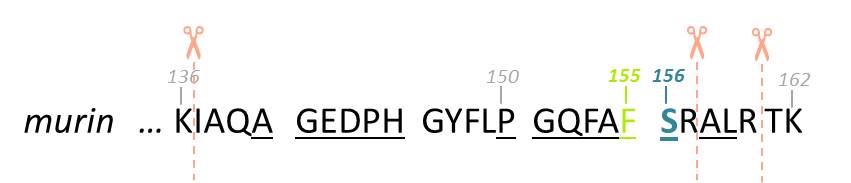

Supplement: Supplementary file 1 — Supplementary file1 (DOCX 34 KB) [file 11010_2022_4430_MOESM1_ESM.docx]
